# Identification of differentially expressed genes and pathways for intramuscular fat deposition in *pectoralis major* tissues of fast-and slow-growing chickens

**DOI:** 10.1186/1471-2164-13-213

**Published:** 2012-05-30

**Authors:** Huan-Xian Cui, Ran-Ran Liu, Gui-Ping Zhao, Mai-Qing Zheng, Ji-Lan Chen, Jie Wen

**Affiliations:** 1 Institute of Animal Sciences, Chinese Academy of Agricultural Sciences, Beijing, 100193, People’s Republic of China; 2 State Key Laboratory of Animal Nutrition, Beijing, 100193, People’s Republic of China

## Abstract

**Background:**

Intramuscular fat (IMF) is one of the important factors influencing meat quality, however, for chickens, the molecular regulatory mechanisms underlying this trait have not yet been determined. In this study, a systematic identification of candidate genes and new pathways related to IMF deposition in chicken breast tissue has been made using gene expression profiles of two distinct breeds: Beijing-you (BJY), a slow-growing Chinese breed possessing high meat quality and Arbor Acres (AA), a commercial fast-growing broiler line.

**Results:**

Agilent cDNA microarray analyses were conducted to determine gene expression profiles of breast muscle sampled at different developmental stages of BJY and AA chickens. Relative to d 1 when there is no detectable IMF, breast muscle at d 21, d 42, d 90 and d 120 (only for BJY) contained 1310 differentially expressed genes (DEGs) in BJY and 1080 DEGs in AA. Of these, 34–70 DEGs related to lipid metabolism or muscle development processes were examined further in each breed based on Gene Ontology (GO) analysis. The expression of several DEGs was correlated, positively or negatively, with the changing patterns of lipid content or breast weight across the ages sampled, indicating that those genes may play key roles in these developmental processes. In addition, based on KEGG pathway analysis of DEGs in both BJY and AA chickens, it was found that in addition to pathways affecting lipid metabolism (pathways for MAPK & PPAR signaling), cell junction-related pathways (tight junction, ECM-receptor interaction, focal adhesion, regulation of actin cytoskeleton), which play a prominent role in maintaining the integrity of tissues, could contribute to the IMF deposition.

**Conclusion:**

The results of this study identified potential candidate genes associated with chicken IMF deposition and imply that IMF deposition in chicken breast muscle is regulated and mediated not only by genes and pathways related to lipid metabolism and muscle development, but also by others involved in cell junctions. These findings establish the groundwork and provide new clues for deciphering the molecular mechanisms underlying IMF deposition in poultry. Further studies at the translational and posttranslational level are now required to validate the genes and pathways identified here.

## Background

During the past decades, meat poultry breeding has been predominantly focused on accelerating growth rate and yields of breast and thigh meat. The impressive progress made in these traits, however, has been accompanied by deterioration of taste quality of the broiler meat and, in some markets, decreased acceptability of the meat by consumers [1,2].

Intramuscular fat (IMF), located in most species in the epimysium, perimysium and endomysium, is an important determinant of meat quality. A certain amount of IMF can enhance meat quality traits such as the flavor, juiciness, water holding capacity and tenderness [3-7]. Additionally, IMF can improve meat quality by reducing the drip loss and cooking loss [8].

Compared to beef, chicken meat does not present a marbled aspect and intramuscular fat is not visible and not anatomically separable; a higher proportion of IMF is represented by polar lipids, presumably within membranes. Previous studies found that the IMF content of chicken meat increased with age from d 28 to d 90 [9,10] and may increase further after that (d 90-d 140), along with flavor and taste of the meat [11].

For livestock IMF, studies of the molecular mechanisms underlying IMF deposition have revealed large numbers of DEGs and signaling pathways including ADAMST4-signaling and insulin-signaling pathways [12-17]. Compared to mammals, where most *de novo* synthesis of fatty acids occurs in adipose tissue [18], chicken differs in lipid metabolism because little fatty acid synthesis occurs in adipose tissues in this species [19-21]. Until now, no systematic studies have been reported on the IMF development in chicken at the molecular level, although expression profiles in abdominal fat tissue [22], liver [23] or muscle cells [24] have been performed. This study provides a comprehensive analysis of gene expression profiles of chicken breast using both fast- and slow-growing breeds.

## Results

### Differentially expressed gene profiles in breasts of slow- and fast-growing chickens

To identify potential candidate genes affecting chicken IMF deposition, gene expression profiles in breast muscle of both Beijing-you (BJY, a slow-growing Chinese breed) and Arbor Acres (AA, commercial fast-growing broiler) chickens at different developmental stages were examined using Agilent cDNA microarray technology. Divergence of breast growth rates in BJY and AA chickens are shown in Table 1. Of the target traits measured, IMF contents and muscle weights at later ages (d 21, d 42, d 90 and d 120 just for BJY) were all higher than the content and weight at d 1 (Table 1). Thus, for each breed, the gene expression profile at d 1 was used as the control and the DEGs analyses were carried out as comparisons with d 1 (21 vs 1, 42 vs 1, 90 vs 1 and 120 vs 1, just for BJY). For AA chickens, 4255 genes (1310 known) were detected as DEGs with consistent fold changes ≥2.0, in all comparisons. In BJY chickens, 3182 genes (1080 known) were detected as DEGs (Table 2). There were 1746 DE genes (595 known) shared by the two breeds, (Additional file 1, Figure 1).

**Table 1:** Changes in IMF, TG, PLIP and breast muscle weight in two chicken breeds at different ages

**Ages and breeds**	**IMF (%)**		**TG (mg/g)**		**PLIP (mg/g)**		**Muscle weight (g)**	
	**BJY**	**AA**	**BJY**	**AA**	**BJY**	**AA**	**BJY**	**AA**
1	0	0	0	0	0	0	1.34 ± 0.17^e^	1.58 ± 0.13^d^
21	1.83 ± 0.13^b^	2.49 ± 0.32^b^	5.19 ± 0.63^b^	10.88 ± 0.54 ^b^	2.67 ± 0.30 ^b^	0.53 ± 0.16 ^b^	7.73 ± 0.59^d^	41.29 ± 1. 29^c^
42	2.43 ± 0.25^a^	2.11 ± 0.36^b^	8.28 ± 0.92 ^a^	8.81 ± 0.72 ^b^	2.82 ± 0.30 ^b^	2.14 ± 0.26 ^a^	15.22 ± 0.78^c^	132.88 ± 2.01^b^
90	2.74 ± 0.15^a^	5.39 ± 0.88^a^	12.13 ± 1.27^a^	22.68 ± 0.65 ^a^	3.90 ± 0.31 ^ab^	3.42 ± 0.29 ^a^	49.67 ± 1.52^b^	351.29 ± 3.89^a^
120	2.67 ± 0.36^a^	/	9.25 ± 1.11^a^	/	5.77 ± 0.30 ^a^	/	96.50 ± 1.29^a^	/

Data are means ± SD (n = 6), different lowercase superscripts in each column indicate significant differences (P < 0.05). Content of lipids (IMF, TG, PLIP) within breast tissue at d 1 were too little to measure by current method and were set to “0”. IMF, Intramuscular fat; TG, Triglyceride; PLIP, Phospholipids; BJY, Beijing-you; AA, Arbor Acres.

**Table 2:** Summary of gene expression in breast muscle of BJY and AA chickens determined by microarray analysis

**Breed**	**Total probes**	**Non-expressed genes**	**Expressed genes**	**Differentially expressed genes**	
				**Known**	**Not-known**
AA	43024	8125	25446	1310	2945
BJY		8046	24749	1080	2102

BJY, Beijing-you; AA, Arbor Acres.

**Figure 1:**
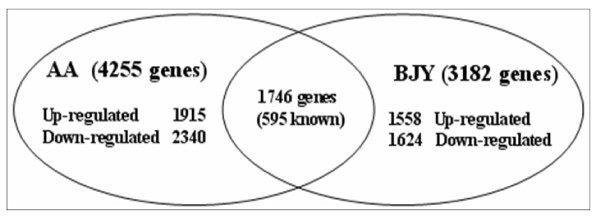
**Numbers of genes that were differentially expressed in breast muscle between days 21, 42, 90 and 120 (BJY) versus day 1.** BJY, Beijing-you; AA, Arbor Acres.

Based on the known DE genes, GO analysis was performed in each breed, and the enriched GO-terms (P < 0.05) in the ontology classification “Biological Process” were selected and are presented in Additional file 2. The results showed the consistency of enriched GO-terms between the two breeds, mainly including the following processes: muscle system, lipid metabolism, cell cycle, protein metabolism, hormone metabolism, trans-membrane transport, oxidation-reduction, regulation of cell differentiation, regulation of immune system, blood circulation, regulation of apoptosis and ATP biosynthesis.

**Figure 2:**
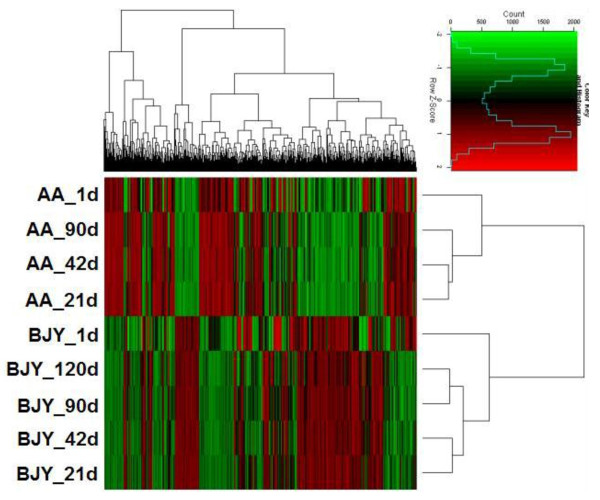
**Cluster analysis of all microarrays.** Expression profiles of samples at different ages (1d, 21d, 42d, 90d and 120d marked) of the same breed (AA and BJY marked) were polymerized together; the expression profile of genes at d 1 differed more than those at other ages. BJY, Beijing-you; AA, Arbor Acres.

To validate the microarrays, normal distribution analysis was performed with the results of the nine microarrays, and all of their coefficients were <0.5. Based on the 595 known genes that were shared as DEGs in both breeds, cluster analysis of all microarrays was performed (Figure 2) using the Cluster 3.0 software. The results demonstrated that expression profiles of samples at different ages of the same breed were polymerized together; the expression patterns of genes at d 1 differed more than those at other ages.

To further validate the results of microarray testing, q-PCR was used to examine the relative expression of 9 DE key genes selected in each breed at different ages. The results showed acceptable consistency between the results of q-PCR and the fold-change of DE genes in the microarrays (Table 3).

**Table 3:** Comparison of microarray and q-PCR fold-changes for selected transcripts in breast muscle of BJY or AA chickens

**Breed**	**Comparison (days)**	**21 vs 1**		**42 vs 1**		**90 vs 1**		**120 vs 1**	
		**Microarray**	**q-PCR**	**Microarray**	**q-PCR**	**Microarray**	**q-PCR**	**Microarray**	**q-PCR**
*BJY*	FABP3	−5.86	−5.99	−3.56	−5.44	−7.90	−6.51	−2.38	−3.78
	RXRA	3.64	2.63	4.11	5.44	5.83	9.38	3.08	6.78
	MYL10	−152.02	−20.08	−87.27	−12.47	−291.39	−69.91	−141.24	−33.06
	FGF4	4.82	3.66	4.24	4.69	11.43	8.55	6.55	9.99
	RBP7	4.89	3.56	9.27	6.42	7.14	9.71	2.47	3.53
	ACSF3	−2.40	−2.92	−3.98	−3.26	−4.49	−4.61	−3.86	−3.77
	DCK	−2.49	−2.18	−11.36	−7.45	−19.25	−9.19	−44.86	−12.93
	DGAT2	5.38	5.59	2.26	3.24	2.20	2.57	2.30	2.69
	FABP5	−2.68	−3.11	−3.57	−3.42	−2.97	−3.07	−3.47	−3.78
*AA*	FABP3	−4.62	−4.56	−4.78	−3.77	−5.01	−7.39	/	/
	RXRA	2.86	2.5	4.51	4.79	3.48	3.69	/	/
	MYH4	−30.21	−8.48	−50.39	−12.56	−29.66	−13.53	/	/
	FGF4	−11.24	−12.54	−9.05	−8.67	−3.60	−4.22	/	/
	FABP1	12.81	6.97	5.21	4.33	2.23	3.15	/	/
	PGK1	7.44	6.98	8.13	7.82	10.04	9.53	/	/
	PLTP	3.62	3.06	2.92	2.45	6.67	4.36	/	/
	RBP7	−8.04	−9.41	−12.33	−10.95	−2.50	−3.27	/	/
	THRSP	−8.45	−7.99	−6.29	−5.46	−35.01	−19.74	/	/

FABP (1, 3, 5), Fatty acid binding protein; RXRA, Retinoid X receptor-alpha; MYL10, Myosin, light chain 10; FGF4, Epidermal growth factor 4; RBP7, Retinol binding protein 7; ACSF3, Acyl-CoA synthetase family member 3; DCK, Deoxycytidine kinase; DGAT2, Diacylglycerol O-acyltransferase homolog 2; MYH4, Myosin, heavy polypeptide 4; PGK1, Phosphoglycerate kinase 1; PLTP, Phospholipid transfer protein; THRSP, Thyroid hormone responsive, SPOT14 homolog; BJY, Beijing-you; AA, Arbor Acres.

### Key genes related to lipid metabolism and muscle development

As IMF is located throughout skeletal muscle, and not as discrete adipose deposits in chicken, it is reasonable to assume that DEGs related to muscle development or lipid metabolism in current study would contribute to its process of deposition. The GO-term analysis showed that known DE genes related to muscle development (34 in BJY, 39 in AA) and genes related to lipid metabolism (59 in BJY, 70 in AA) were found (Additional file 3, Additional file 4). The 19 DE genes related to muscle development that were shared by the two breeds included *FGF*4, *MYH*4, *MYBPC*1, *MYH*7*B*, *MYL*2, *MYL*10, *MYL*3, *MYO*15*A*, *TGFA* and *WNT*4 (Additional file 5); these might be key genes. The 33 DEGs affecting lipid metabolism that were shared by BJY and AA breeds included *ACSL*1, *ACSS*2, *APOH*, *FABP*1, *FABP*3, *CETP*, *RXRA*, *PPARGC*1*A*, *SNX*4 and *SNX*30 (Additional file 5).

Genes for which the expression was significantly correlated with changes in muscle development and lipid deposition were analyzed as these genes might be directly involved in IMF regulation. The changes in muscle weight, the content of IMF, triglyceride (TG) and phospholipids (PLIP) in breast tissue of the two breeds at different ages are provided in Table 1. The striking difference between the breeds in the rate of growth of the breast muscle is apparent but growth continues across all ages sampled.

Based on the trait measurements, relationships between expression of DEGs and muscle development and lipid deposition (IMF, TG, PLIP contents) were examined. The expression of *GDF*3 and *CAPNS*1 in AA, and *ANKRD*1 and *PLG* in BJY were positively correlated (p < 0.05) with muscle development. The expression of *BMP*2*K* and *MYBPC1* in AA, and *CENPF*, *ELN*, *FGF*7, *FGFR*1, *MYBL*2, *MYCN* and *MYBPC*1 in BJY were negatively correlated (p < 0.05) with that of muscle development (Table 4). For lipid deposition, *CH*25*H*, RCJMB04_10b24, RCJMB04_13o20, *SNX*3, and *CETP* in AA, and *YWHAH*, *NR*3*C*2 and *CETP* in BJY were positively correlated (p < 0.05) with PLIP contents while *NACA*, *RBP*7 and *GLTPD*1 in AA chickens, and *LOC416618*, *ETFDH* and *GLTPD*1 in BJY chickens were negatively correlated (p < 0.05) with PLIP contents. In the case of TG deposition, *HMGCLL*1, *THBS*1, *UCP*3 and *SNX*4 in AA, and *EHHADH* and *SNX*4 in BJY were positively correlated (p < 0.05) with TG contents and *SGPL*1, *SH*3*PXD*2*B* and *THRSP* in just the AA chickens were negatively correlated (p < 0.05) with contents of TG or IMF (Table 4). The expression of 1 DEG (*MYBPC*1), common to both breeds, was positively correlated with breast tissue weight across ages while 3 DEGs (*CETP*, *GLTPD*1 and *SNX*4), common to the two breeds, were positively or negatively correlated with IMF, TG or PLIP contents (Table 4).

**Table 4:** **Key genes**^**1**^**related to breast muscle development or lipid metabolism in two breeds of chickens**

**Breed**	**Symbol**	**Gene**	**Coefficient**	**Regulation**	**Trait**
**AA**	BMP2K	BMP-2-inducible protein kinase	−0.97	-	Breast muscle
	CAPNS1	Calpain, small subunit 1	1.00	+	
	GDF3	Growth differentiation factor 3	1.00	+	
	CH25H	Cholesterol 25-hydroxylase	0.92	+	PLIP
	HMGCLL1	3-hydroxymethyl-3-methylglutaryl- Coenzyme A lyase-like 1	0.97 or 0.97	+	TG or IMF
	NACA	Nascent polypeptide-associated complex alpha subunit	−1.00	-	PLIP
	RBP7	Retinol binding protein 7	−0.95	-	PLIP
	RCJMB04 _10b24	membrane bound O-acyltransferase domain containing	1.00	+	PLIP
	RCJMB04 _13o20	NSFL1 (p97) cofactor (p47)	1.00	+	PLIP
	SGPL1	Sphingosine-1-phosphate lyase 1	−1.00 or −1.00	-	TG or IMF
	SH3PXD2B	SH3 and PX domains 2B	−0.95 or −0.96	-	TG or IMF
	SNX3	Sorting nexin 3	0.96	+	PLIP
	THBS1	Thrombospondin-1	1.00 or 1.00	+	TG or IMF
	THRSP	Thyroid hormone responsive, SPOT14 homolog	−0.86 or −0.85	-	TG or IMF
	UCP3	Uncoupling protein 3	1.00 or 1.00	+	TG or IMF
**BJY**	ANKRD1	Ankyrin repeat domain 1	0.94	+	Breast muscle
	CENPF	Centromere protein F	−0.77	-	
	ELN	Elastin	−0.92	-	
	FGF7	Fibroblast growth factor 7	−0.99	-	
	FGFR1	Fibroblast growth factor receptor 1	−0.98	-	
	PLG	Plasminogen	0.96	+	
	MYBL2	V-myb myeloblastosis viral oncogene homolog (avian)-like 2	−0.97	-	
	MYCN	V-myc myelocytomatosis viral related oncogene, neuroblastoma derived	−0.94	-	
	YWHAH	Tyrosine 3-monooxygenase/tryptophan 5-monooxygenase activation protein	0.99	+	PLIP
	NR3C2	Nuclear receptor subfamily 3, group C, member 2	0.95	+	PLIP
	LOC416618	NAD(P) dependent steroid dehydrogenase-like	−0.74	-	PLIP
	EHHADH	Enoyl-Coenzyme A, hydratase/3-hydroxyacyl Coenzyme A dehydrogenase	0.90 or 0.85	+	TG or IMF
	ETFDH	Electron-transferring-flavoprotein dehydrogenase	−0.95	-	PLIP
**AA/BJY**^2^	MYBPC1	Myosin binding protein C, slow type	0.96/0.96	+	Breast muscle
	CETP	Cholesteryl ester transfer protein	0.98/0.80	+	PLIP
	GLTPD1	Glycolipid transfer protein domain containing 1	−1.00/-0.96	-	PLIP
	SNX4	Sorting nexin 4	0.95/1.00 or 0.94/0.90	+	TG or IMF

All correlations were significant (p < 0.05 or p < 0.01). ^1^Key gene were defined as its expression determined by microarray was significantly correlated (p < 0.05 or p < 0.01) with changes in breast muscle weight or in content of IMF, TG or PLTP across the sampled ages. ^2^Common to both BJY and AA breeds, the expression of genes was significantly correlated with target traits across ages. “+” represents up-regulated effect; “-” represents down-regulated effect. d = day. IMF, Intramuscular fat; TG, Triglyceride; PLIP, Phospholipids; BJY, Beijing-you; AA, Arbor Acres.

**Figure 3:**
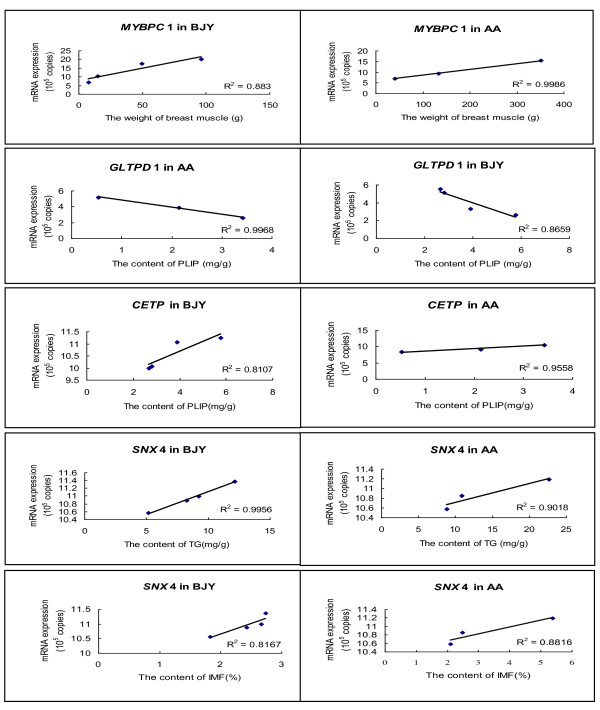
**Correlation analysis between the mRNA expression of MYBPC1 (Myosin binding protein C, slow type), GLTPD1(Glycolipid transfer protein domain containing 1), CETP (Cholesteryl ester transfer protein) or SNX4(Sorting nexin 4) genes determined by q-PCR and the breast muscle weight, PLIP, IMF or TG contents in BJY or AA chickens.** The r value indicates Spearman’s Correlation. IMF, Intramuscular fat; TG, Triglyceride; PLIP, Phospholipids; BJY, Beijing-you; AA, Arbor Acres.

The relative abundances of *MYBPC*1, *GLTPD*1, *CETP, SNX*4 transcripts in BJY chickens and in AA chickens were further measured by q-PCR. Consistent correlation between mRNA expression of these genes and muscle development and lipid deposition (IMF, TG, PLIP contents) were found and are shown in Figure 3.

### Pathways and a regulatory network for IMF content in chickens

**Figure 4:**
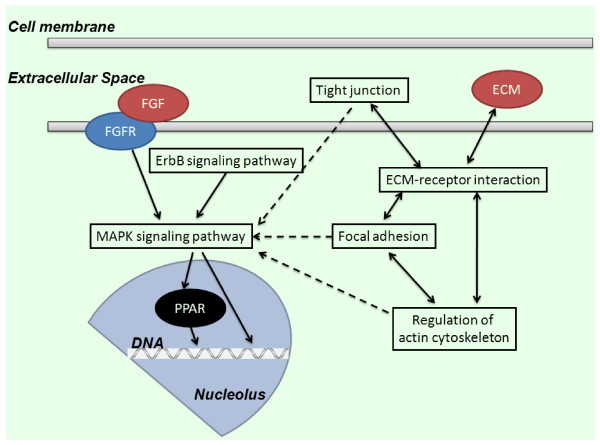
**Proposed regulatory network for chicken IMF based on significantly different GO terms and KEGG pathways.** This network is involved in several cellular functions including lipid metabolism (pathways for ErbB, MAPK & PPAR signaling) and cell junction (tight junction, ECM-receptor interaction, focal adhesion, regulation of actin cytoskeleton). Whether or not cell junction related pathways function on IMF deposition through lipid metabolism related pathways (dotted lines) needs further study. FGF & FGFR, Fibroblast growth factor and its receptor; PPAR, Peroxisome proliferator-activated receptor; ECM, Extracellular matrix.

The regulation of IMF is possibly a function of complex pathway interactions involving muscle, fat and connective tissue [25], so examining regulatory networks is the preferred method of analysis. After KEGG pathway analysis of the known DEGs related to muscle development and lipid metabolism, 24 metabolic pathways were identified in each breed, with 15 being shared by BJY and AA chickens (Additional file 6). Well known pathways affecting lipid metabolism (MAPK- and PPAR-signaling) were enriched in both breeds; the ErbB signaling pathway probably plays a role upstream of MAPK signaling. These analyses also demonstrate that pathways related to cell junctions (tight junction, ECM-receptor interaction, focal adhesion, regulation of actin cytoskeleton) were also enriched and might form a network with pathways related to lipid metabolism to influence the deposition of IMF (Figure 4).

## Discussion

### cDNA array analysis

Fat deposition in chickens takes place mainly in visceral adipose tissue and in muscle. The latter, IMF, is very important for sensory aspects of meat quality and there is increasing interest in improving quality, perhaps using marker-assisted selection for IMF. Although global gene expression surveys have been performed on visceral tissues [22], this study is the first to systematically explore gene expression profiles in breast tissue using two distinct breeds across their development. The present objective was to identify global genes and pathways affecting chicken IMF deposition.

**Figure 5:**
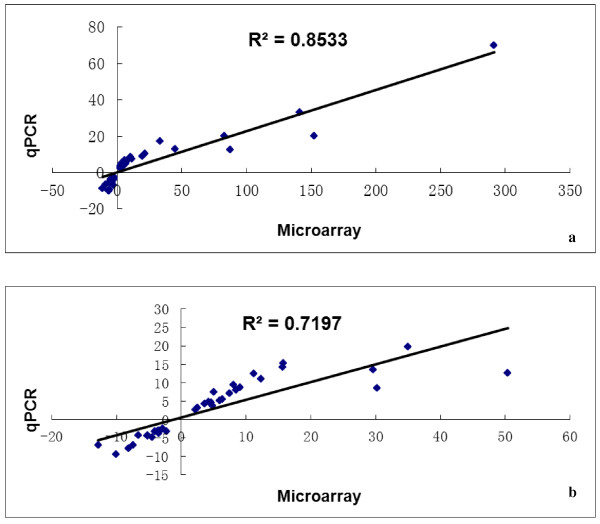
**Technical validation of microarray results by q-PCR by correlation: (a) BJY and (b) AA chickens.** The r value indicates Spearman’s Correlation between the two methods.

Chicken microarrays were employed, each using pooled RNA samples (n = 6 birds, within each of two breeds and at 4 or 5 ages; 9 arrays in all). Such a pooling strategy can dramatically improve accuracy when only one array is available in each biological condition [26]. Potential candidate DEGs related to IMF deposition were rigorously defined, requiring their expression to differ across all comparisons conducted (21 vs 1, 42 vs 1, 90 vs 1 and 120 vs 1, just for BJY). To confirm results from the microarrays, more than 100 tests were done with q-PCR, involving 12–13 genes in breast tissue at 4 or 5 sampled ages in each breed. As shown in Figure 5, fold-changes in gene expression between the two methods were correlated in both BJY (r^2^ = 0.85) and AA (r^2^ = 0.72) chickens.

### Function of key DEGs affecting IMF and muscle development

The data obtained here indicate that three key genes related to lipid metabolism were shared by the two breeds (*CETPGLTPD*1 and *SNX*4) and showed consistent trends with the changes in IMF, TG or PLIP contents across the ages examined (Table 4). Cholesteryl ester transfer protein (CETP) functions in the reversible transport/exchange of cholesteryl esters from high-density lipoproteins (HDL) and triglycerides from very-low-density (VLDL) or low-density (LDL) lipoproteins. The *SNX*4 gene encodes a member of the sorting nexin family which associates with a variety of receptors, including those for insulin, EGF and leptin [27]. Glycolipid transfer protein D1 (GLTPD1) is a cytosolic protein that transfers glycolipids between different intracellular membranes [28]; its precise biological function is not known.

For key candidate genes affecting muscle development, one gene common to the two breeds (*MYBPC1*) changed consistently with the changing patterns of breast tissue weight (Table 4). The *MYBPC*1 gene encodes a slow form(s) of MyBP C found in skeletal muscle and functions in the assembly and stabilization of sarcomeric M- and A-bands and regulates the contractile properties of actomyosin filaments. In chickens, increased levels of MyBP-C slow are found in dystrophic skeletal muscles [29]. Further research is required to understand the molecular mechanisms that lead to the anticipated effects of this candidate gene on lipid metabolism and muscle development in chickens.

### Function of novel pathways related to IMF

GO-term analysis was used to explore the function of DEGs and KEGG pathway analysis was used to explore the regulatory network underlying chicken IMF deposition. As expected, several well-known pathways related to lipid metabolism were found, including the MAPK and PPAR signaling pathways [30]. Large numbers of DEGs involved in PPAR signaling pathways here have been proven to be functional in lipid metabolism, such as FABP family genes (*FABP*1, *FABP*5, *FABP*6), *ACSL*4, *CD*36, *PLTP*[31-33]. This is partially consistent with our previous studies where mRNA expression of *adipocyte FABP* and *heart-type FABP* genes and SNP markers from these genes were found to be associated with IMF content in Chinese chickens [34,35]. Several DEGs (*FGF*1, *FGF*4, *FGF*7, *FGF*16, *FGFR*1 and *FGFR*2) belonging to the FGF family and receptors were reflected in the MAPK signaling pathway.

Of special interest, these GO and KEGG analyses provide the first demonstration that a series of pathways related to cell junctions might contribute to the deposition of IMF. DEGs related to muscle development included well-known genes (*MYBPC*1, *MYBPC*2, *MYH*4, *MYH*7*BMYL*10, *MYL*2, *MYL*3, *MYO*15*A* and *MY0*1*F*) related to the biosynthesis of myosin, of which *MYL*10 and *MYL*2 encode proteins involved in the formation of tight junctions, focal adhesions and regulation of the actin cytoskeleton. Additionally, the *CTNNB*1 gene encodes β-catenin, one of the proteins constituting adherens junctions, and anchoring the actin cytoskeleton [36,37]; *ACTB* encodes beta-actin, one of the non-muscle cytoskeletal actins playing a central role in shape determination, cytokinesis, and cell motility, along with cell-cell and cell-matrix interactions [38,39]. In addition, DEGs of the FGF family and their receptors (*FGF*1, *FGF*4, *FGF*7, *FGF*16, *FGFR*1 and *FGFR*2) were reflected in the regulation of the actin cytoskeleton and may modulate morphogenetic processes involving cellular rearrangements and tissue remodeling [40,41]. The genes *THBS*1 and *CD*36 are involved in extracellular matrix (ECM)-receptor interaction [42,43] and influence, directly or indirectly, cellular activities such as adhesion and migration. In support of this, previous studies have shown that changes in cytoskeletal organization and its contacts with the ECM are essential in the morphogenesis of fibroblastic preadipocytes to rounded, mature adipocytes [44], while the expression of actin, integrins and several cytoskeletal proteins is down-regulated during adipogenesis [45,46]. Taken together, cell junctions including the interaction of the ECM and cytoskeleton might participate in accumulation of IMF during chicken development.

The KEGG analysis implicated the MAPK signaling pathway in processes involving tight junctions, focal adhesion and regulation of the actin cytoskeleton. This is also consistent with studies showing that activation of MAPK activity resulted in the disruption of tight junctions, and that inhibition of MAPK activation prevented this process [47,48]. We suggest that processes related to cell junctions might interact with pathways related to lipid metabolism, mainly through MAPK activity, to influence the deposition of IMF. The proposed molecular regulatory network affecting IMF deposition during chicken development is presented in Figure 4. This novel suggestion of IMF regulation and its detailed mechanism through pathways related to cell junctions in addition to lipid metabolism needs further examination.

The present approach has used gene expression profiling to elucidate molecular mechanisms of post-hatch IMF deposition in chickens. Possible regulation by translational mechanisms and posttranslational modifications may also contribute. A more complete understanding of IMF development in chickens should include further examination of the expression and function of the proteins encoded by the genes identified here in both embryonic and post-hatch stages of development.

## Conclusions

With aim of identifing global candidate genes and new pathways related to IMF deposition in chicken breast, Agilent cDNA microarray analyses were performed with both fast- and slow- growing breeds. Gene expression profiles of breast muscle sampled at different developmental stages of BJY and AA chickens were determined. Relative to d 1, breast muscle at d 21, d 42, d 90 and d 120 (only for BJY) contained 1310 DEGs in BJY and 1080 DEGs in AA. Several DEGs (*MYBPC*1*, CETP*, *GLTPD*1 and *SNX*4) may play key roles in IMF developmental processes because their expressions were correlated with the changing patterns of lipid content or breast weight across the ages sampled in both two breeds. In addition, the results of KEGG pathway analysis imply that IMF deposition in chickens is regulated and mediated not only by genes and pathways related to lipid metabolism and muscle development, but also by others involved in cell junctions with the function in maintaining the integrity of tissues and signal transduction. These findings establish the groundwork and provide new clues for deciphering the molecular mechanisms underlying IMF deposition in poultry. Additional studies of translational and posttranslational effects will be required to complement these mRNA expression analyses.

## Methods

### Animals

All experimental procedures, using female Beijing-You chickens (BJY, the Institute of Animal Sciences, Chinese Academy of Agricultural Sciences, Beijing, China) and Arbor Acres (AA, Dadongliu broiler Company, Beijing, China), were performed in accordance with the Guidelines for Experimental Animals established by the Ministry of Science and Technology (Beijing, China). Individuals within each breed had the same genetic background. Birds (60 BJY and 48 AA) were reared in stair-step caging under continuous lighting using standard conditions of temperature, humidity and ventilation. Chickens used for sample collection at d 1 were not fed. The same diet was fed to all chickens and was formulated to be intermediate between recommendations for the two breeds [49,50]. The starter ration (d 1 to d 21) with 20% crude protein and 2.87 MC/kg differed only slightly from that used in the grower (after d 22) phase; 19% crude protein and 3.0 MC/Kg. Feed and water were provided *ad libitum* during the experiment.

### Sample collection

At each sampling age, d 1, d 21, d 42, d 90, and d 120 (only for BJY), six birds of similar weight from each breed were sacrificed for tissue collection. Samples of the left *pectoralis major* muscle were excised, snap-frozen in liquid nitrogen and stored at −80°C. The entire right breast was collected and stored at −20°C for trait measurements.

### Trait measurements

Intramuscular fat (IMF) content of breast muscle was determined by extraction with petroleum ether in a Soxhlet apparatus [51,52] and expressed as percentages of the dry weight of the muscle.

Samples of the right *pectoralis major* muscle were homogenized using the method of Folch [53]. The contents of triglyceride (TG) and phospholipids (PLIP) in the solvent phase, after centrifugation, were analyzed with TG [54,55] and PLIP [56,57] kits (Deliman Biochemical technology Co., LTD, Beijing, China).

### Total RNA preparation and microarray hybridization and analysis

Total RNA was isolated from breast muscle samples using Trizol reagent (Invitrogen, USA) according to the manufacturer's instructions and dissolved in RNase-free water at a final 2.0 μg/μl concentration. RNA from *Pectoralis major* collected at each sampling age were extracted, and pooled within days and breeds for testing with microarrays. Microarray hybridization was carried out by GeneTech Biotechnology Limited Company (Shanghai, China) using Agilent Chicken Gene Chips (ID: 015068) with 42034 probes. Array scanning and data extraction were carried out following the standard protocol.

The normal distribution of signal plots in every chip was determined. Clustering was performed based on the DE genes in each chicken breed, using un-centered Pearson correlations and average linkage cluster 3.0, and was displayed in TreeView. Normalized fluorescence intensity values of each dye-swapped experiment were averaged separately for sample and reference channels. Thereafter, for each probe, averaged sample and reference fluorescence values were log2-transformed. Average linkage hierarchical clustering was performed using the Euclidian metric. In heat-maps, the color of features (probes) was determined by log2 (reference/sample).

### Analysis of gene expression profile and differentially expressed genes

The distribution of expressed genes was analyzed by JMP4.0 according to their expression level. If the flag of a gene was “A” by the scanner according to the data normalization and results of Agilent Microarray Suite 4.0 software, it was considered to be "not detected", and hence "not expressed" in this study. Similarly, the genes with “P” flags were considered to be “expressed transcripts”. Expressed transcripts were defined as being present in samples of at least one sampled age and were used for all following studies. The expression value of each probe set was normalized and calibrated using the RMA method.

Screening of differentially expressed genes (DEGs) was performed on the basis of differences in the IMF, TG and PLIP contents at different ages in each breed. Expression at d 1 was used as the controls, and comparisons were made within each breed at d 21, 42, 90 and 120 (only for BJY). Genes were considered to be DEGs only when the fold-change in abundance for all comparisons exceeded 2.0.

### Quantitative real time RT-PCR (q-PCR)

To avoid amplification of any residual genomic DNA, all PCR primers were placed at or just outside the exon/exon junctions and specificity was determined with BLASTN (Additional file 7). After a general reverse transcription reaction, PCR analyses were performed in 20 ul amplification reactions containing 10 ul of 2× SYBR Green PCR Master Mix (Tiangen Biological Technology Co., Ltd, Beijing, China), 20 ng cDNA and 0.5 μl (10 mM) of each primer using the following conditions according to the manufacturer's instructions: 95°C for 10 minutes for 1 cycle, 40 cycles at 95°C for 15 seconds and then at 63°C for 45 seconds.

Quantification of the transcripts was determined using standard curves with 10-fold serial dilutions of cDNA (10^-7^ to 10^-12^ g). Melting curves were constructed to verify that only a single PCR product was amplified. Within runs, samples were assayed in triplicate with standard deviations of threshold cycle (CT) values not exceeding 0.5, and each q-PCR run was repeated at least twice. Negative (without template) reactions were performed within each assay.

### Gene ontology enrichment analysis and visualization

Gene Ontology enrichment analysis was performed for features corresponding to DEG in each breed using the GOEAST software toolkit. The significance level of GO term enrichment was set as FDR-adjusted p-value less than 0.05 by the Yekutieli method.

### Screening of the key DE genes

Correlation analysis was performed between the key DE genes related to muscle development or lipid metabolism with changes in lipid content or breast muscle weight across the sampled ages within each breed. If the expression of a given gene was significantly correlated (p < 0.05) with breast tissue weight, or content of IMF, TG or PLIP, this gene was considered to be a key DE gene.

### KEGG pathway analysis

KEGG pathway [58-60] information was used in this analysis. Probeset IDs of each category were first mapped to NCBI Entrez gene IDs according to the Agilent Chicken Array annotation file, and then were mapped to KEGG gene IDs according to the KEGG gene cross-reference file. Pathways that were significantly enriched with DEGs were identified by a hypergeometric test using R packages (p < 0.01, FDR adjusted). Pathways with < 3 known chicken genes were discarded. Graphical pathway maps were downloaded from the KEGG FTP server, and DEGs were then highlighted in them according to the coordinate description in the XML files at the KEGG FTP server, using Perl GD, XML:Parser and XML:LibXML modules.

## Abbreviations

ACSF3, Acyl-CoA synthetase family member 3; ACSL1, Acyl-CoA synthetase long-chain family member 1; ACSS(1&2), Acyl-CoA synthetase short-chain family member; ACTB, Actin, beta; ADAMST4, A disintegrin and metalloproteinase with thrombospondin motif 4; ANKRD1, Ankyrin repeat domain 1; APOH, Apolipoprotein H; BMP(4,7), Bone morphogenetic protein; BMP2K, BMP-2-inducible protein kinase; CAMs, Cell adhesion molecules; CAPNS1, calpain, small subunit 1; CD36, Cluster of Differentiation 36; CD44, CD44-like protein; CENPF, Centromere protein F; CETP, Cholesteryl ester transfer protein; CH25H, Cholesterol 25-hydroxylase; CTNNB1, Catenin (cadherin-associated protein), beta 1; CYP17A1, Cytochrome P450, family 17, subfamily A, polypeptide 1; DCK, Deoxycytidine kinase; DGAT2, Diacylglycerol O-acyltransferase homolog 2; ECM, Extracellular matrix; EGF, Epidermal growth factor; EHHADH, Enoyl-Coenzyme A, hydratase/3-hydroxyacyl Coenzyme A dehydrogenase; ELN, Elastin; ErbB, Erythroblastic Leukemia Viral Ongene Homolog; ETFDH, Electron-transferring-flavoprotein dehydrogenase; FABP(13, 5, 6, A/Adipocyte, H/Heart), Fatty acid binding protein; FGF(47, 16), Fibroblast growth factor; FGFR, Fibroblast growth factor receptor; GDF3, Growth differentiation factor 3; GLTPD1, Glycolipid transfer protein domain containing 1; HMGCLL1, 3-hydroxymethyl-3-methylglutaryl-Coenzyme A lyase-like 1; KEGG, Kyoto Encyclopedia of Genes and Genomes; LDLR, Low density lipoprotein receptor; LOC416618, NAD(P) dependent steroid dehydrogenase-like,; MAP2K3, Mitogen-activated protein kinase kinase 3; MAPK, Mitogen-activated protein kinase; MBOAT2, Membrane bound O-acyltransferase domain containing 2; MYBL2, V-myb myeloblastosis viral oncogene homolog (avian)-like 2; MYBPC1, Myosin binding protein C, slow type; MYCN, V-myc myelocytomatosis viral related oncogene, neuroblastoma derived; MYF5, Myogenic factor 5; MYH(4, 6, 7B), Myosin, heavy polypeptide; MYL(23, 4, 10), Myosin, light chain; MYO15A, Myosin XVA; NACA, Nascent polypeptide-associated complex alpha subunit; NR3C2, Nuclear receptor subfamily 3, group C, member 2; PGK1, Phosphoglycerate kinase 1; PLTP, Phospholipid transfer protein; PLG, Plasminogen; PPAR, Peroxisome proliferator-activated receptor; PPARGC1A, Peroxisome proliferator-activated receptor gamma, coactivator 1 alpha; RBP7, Retinol binding protein 7; RCJMB04_13o20, NSFL1 (p97) cofactor (p47); RXRA, Retinoid X receptor-alpha; SGPL1, Sphingosine-1-phosphate lyase 1; SH3PXD2B, SH3 and PX domains 2B; SNX(34, 17, 30), Sorting nexin; TGFA, Transforming growth factor, alpha; THBS1, Thrombospondin-1; THRSP, Thyroid hormone responsive, SPOT14 homolog; UCP3, Uncoupling protein 3; YWHAH, Tyrosine 3-monooxygenase/tryptophan 5-monooxygenase activation protein; WNT4, Wingless-type MMTV integration site family, member 4.

## Supplementary Material

Additional file 1 Annotation and changing of 1746 common DE genes in AA and BJY chickens. Click here for file

Additional file 2 Common Enriched GO terms among the differentially expressed genes in both AA and BYJ chickens. Click here for file

Additional file 3 Key genes related to lipid metabolism and muscle development in BJY chicken. Click here for file

Additional file 4 Key genes related to lipid metabolism and muscle development in AA chicken. Click here for file

Additional file 5 Common DE genes related to muscle development or lipid metabolism in AA and BJY. Click here for file

Additional file 6 Identification of enriched KEGG pathways based on known DEGs related to muscle development and lipid metabolism in AA and BJY chickens. Click here for file

Additional file 7 Selected qPCR primer sequences and accession numbers. Click here for file

## References

[B1] Du M Yin J Zhu MJ Cellular signaling pathways regulating the initial stage of adipogenesis and marbling of skeletal muscle Meat Sci 2010 86 1 103 109 10.1016/j.meatsci.2010.04.027 20510530

[B2] Berri C Wacrenier N Millet N Bihan-Duval EL Effect of selection for improved body composition on muscle and meat characteristics of broilers from experimental and commercial lines Poult Sci 2001 80 7 833 838 11469641 10.1093/ps/80.7.833

[B3] Bejerholm C Barton-Gade PA Proceeding of the 32nd European Meeting of Meat Research Workers Effect of intramuscular fat level on eating quality of pig meat 1986 Belgium, Vol. II, Ghent 389 391

[B4] DeVol DL MeKeith FK Bechtel PJ Novakofski J Shanks RD Carr TR Variations in composition and palatability traits and relationship between muscle characteristics and palatability in a random sample of pork carcass J Anim Sci 1988 66 385 395

[B5] Eikelenboom G Hoving-Bolink AH Wal PG The eating quality of pork: 2. The influence of intramuscular fat Fleischwirtschaft 1996 76 517 518

[B6] Fernandez X Monin G Talmant A Mourot J Lebret B Influence of intramuscular fat content on the quality of pig meat-1. Composition of the lipid fraction and sensory characteristics of m. longissimus lumborum Meat Sci 1999 53 59 65 10.1016/S0309-1740(99)00037-6 22062933

[B7] Farmer LJ Richardson RI, Mead GC Poultry Meat Science Poultry meat flavor 1999 CABI publishing, Wallingford 127 158

[B8] Gerbens F Verburg FJ Van Moerkerk HT Engel B Buist W Veerkamp JH te Pas MF Associations of heart and adipocyte fatty acid binding protein gene expression with intramuscular fat content in pigs J Anim Sci 2001 79 2 347 354 11219443 10.2527/2001.792347x

[B9] Chen JL Wen J Wang SB Zhao GP Zheng MQ Li XH Studies on the characteristics of deposition of chicken IMP and IMF Acta Vet Zootech Sin (Chinese) 2005 36 8 843 845

[B10] Chang GB Lei LL Zhang XY Wang KH Chen R Luan DQ Chen GH Development rule of intramuscular fat content in chicken J Anim Vet Adv 2010 9 2 297 298

[B11] Sun HX Tian Y He HX Wang JD Review of the factors and genes on intramuscular fatty acid Prog Vet Med 2006 27 11 49 53

[B12] Moreno SN Rueda J Carabaño MJ Reverter A McWilliam S González C Díaz C Skeletal muscle specific genes networks in cattle Funct Integr Genomics 2010 10 4 609 618 10.1007/s10142-010-0175-2 20524025 PMC2990504

[B13] Lee SH Gondro C van der Werf J Kim NK Lim DJ Park EW Oh SJ Gibson JP Thompson JM Use of a bovine genome array to identify new biological pathways for beef marbling in Hanwoo (Korean Cattle) BMC Genomics 2010 9 11 623 634 21062493 10.1186/1471-2164-11-623 PMC3018137

[B14] Wang YH Bower NI Reverter A Tan SH De Jager N Wang R McWilliam SM Cafe LM Greenwood PL Lehnert SA Gene expression patterns during intramuscular fat development in cattle J Anim Sci 2009 87 1 119 130 18820161 10.2527/jas.2008-1082

[B15] Cánovas A Quintanilla R Amills M Pena RN Muscle transcriptomic profiles in pigs with divergent phenotypes for fatness traits BMC Genomics 2010 11 11 372 387 20540717 10.1186/1471-2164-11-372 PMC2894043

[B16] Kim NK Park HR Lee HC Yoon D Son ES Kim YS Kim SR Kim OH Lee CS Comparative studies of skeletal muscle proteome and transcriptome profilings between pig breeds Mamm Genome 2010 21 5–6 307 319 20532784 10.1007/s00335-010-9264-8

[B17] Zhao X Mo DL Li AN Gong W Xiao SQ Zhang Y Qin LM Niu YN Guo YX Liu XH Cong PQ He ZY Wang C Li JQ Chen YS Comparative Analyses by Sequencing of Transcriptomes during Skeletal Muscle Development between Pig Breeds Differing in Muscle Growth Rate and Fatness PLoS One 2011 6 5 e19774 1–18 10.1371/journal.pone.0019774 21637832 PMC3102668

[B18] Romsos DR Allee GL Leveille GA In vivo cholesterol and fatty acid synthesis in the pig intestine Proc Soc Exp Biol Med 1971 137 570 573

[B19] Leveille GA Glycogen metabolism in meal-fed rats and chicks and the time sequence of lipogenic and enzymatic adaptive changes J Nutr 1967 90 449 460 4380947 10.1093/jn/90.4.449

[B20] Leveille GA In vitro hepatic lipogenesis in the hen and chick Comp Biochem Physiol 1969 28 431 435 10.1016/0010-406X(69)91357-7 5777391

[B21] Pearce J Some differences between avian and mammalian biochemistry Int J Biochem 1977 8 269 275 10.1016/0020-711X(77)90132-X

[B22] Wang HB Li H Wang QG Zhang XY Wang SZ Wang YX Wang XP Profiling of chicken adipose tissue gene expression by genome array BMC Genomics 2007 27 8 193 207 17594506 10.1186/1471-2164-8-193 PMC1914355

[B23] Bourneuf E Hérault F Chicault C Carré W Assaf S Monnier A Mottier S Lagarrigue S Douaire M Mosser J Diot C Microarray analysis of differential gene expression in the liver of lean and fat chickens Gene 2006 372 162 170 16513294 10.1016/j.gene.2005.12.028

[B24] Zheng Q Zhang Y Chen Y Yang N Wang XJ Zhu D Systematic identification of genes involved in divergent skeletal muscle growth rates of broiler and layer chickens BMC Genomics 2009 22 10 87 99 19232135 10.1186/1471-2164-10-87 PMC2656524

[B25] Hocquette JF Gondret F Baeza E Medale F Jurie C Pethick DW Intramuscular fat content in meat-producing animals: development, genetic and nutritional control, and identification of putative markers Animal 2010 4 303 319 10.1017/S1751731109991091 22443885

[B26] Kendziorski C Irizarry RA Chen KS Haag JD Gould MN On the utility of pooling biological samples in microarray experiments Proc Natl Acad Sci USA 2005 102 12 4252 4257 10.1073/pnas.0500607102 15755808 PMC552978

[B27] Haft CR de la Luz Sierra M, Barr VA, Haft DH, Taylor SI: Identification of a family of sorting nexin molecules and characterization of their association with receptors Mol Cell Biol 1998 18 12 7278 7287 9819414 10.1128/mcb.18.12.7278 PMC109309

[B28] Mattjus P Glycolipid transfer proteins and membrane interaction Biochim Biophys Acta 2009 1788 1 267 272 10.1016/j.bbamem.2008.10.003 19007748

[B29] Obinata T Shinbo K Slow-type C-protein in dystrophic chicken fast pectoralis muscle Muscle Nerve 1987 10 4 351 358 10.1002/mus.880100412 3587270

[B30] Kokta TA Dodson MV Gertler A Hill RA Intercellular signaling between adipose tissue and muscle tissue Domest Anim Endocrinol 2004 27 4 303 331 10.1016/j.domaniend.2004.05.004 15519037

[B31] Abumrad NA el-Maghrabi MR Amri EZ Lopez E Grimaldi PA Cloning of a rat adipocyte membrane protein implicated in binding or transport of long-chain fatty acids that is induced during preadipocyte differentiation. Homology with human CD36 J Biol Chem 1993 268 4 17665 17668 7688729

[B32] Chmurzyńska A The multigene family of fatty acid-binding proteins (FABPs): function, structure and polymorphism J Appl Gene 2006 47 1 39 48 10.1007/BF03194597 16424607

[B33] David M Jiang XC Lagrost L Tall AR The role of plasma lipid transfer proteins in lipoprotein metabolism and atherogenesis J Lipid Res 2009 50 Suppl S201 S206 19023137 10.1194/jlr.R800061-JLR200 PMC2674750

[B34] Li WJ Li HB Chen JL Zhao GP Zheng MQ Wen J Gene expression of heart- and adipocyte-fatty acid-binding protein and correlation with intramuscular fat in Chinese chickens Anim Biotechnol 2008 19 3 189 193 18607791 10.1080/10495390802058319

[B35] Ye MH Chen JL Zhao GP Zheng MQ Wen J Associations of A-FABP and H-FABP markers with the content of intramuscular fat in Beijing-You chicken Anim Biotechnol 2010 21 1 14 24 20024783 10.1080/10495390903328116

[B36] Kemler R From cadherins to catenins: cytoplasmic protein interactions and regulation of cell adhesion Trends Genet 1993 9 9 317 321 10.1016/0168-9525(93)90250-L 8236461

[B37] Yap AS Brieher WM Gumbiner BM Molecular and functional analysis of cadherin-based adherens junctions Annu Rev Cell Dev Biol 1997 13 1 119 146 10.1146/annurev.cellbio.13.1.119 9442870

[B38] Amos LA Amos WB Molecules of the cytoskeleton 1991 Macmillan Education, Basingstoke and London

[B39] Bray D Cell movements 1992 Garland Science, New York

[B40] Hogan BL Morphogenesis Cell 1999 96 2 225 233 10.1016/S0092-8674(00)80562-0 9988217

[B41] Martin GR The roles of FGFs in the early development of vertebrate limbs Genes Dev 1998 12 11 1571 1586 10.1101/gad.12.11.1571 9620845

[B42] Bornstein P Diversity of function is inherent in matricellular proteins: an appraisal of thrombospondin 1 J Cell Biol 1995 130 3 503 506 10.1083/jcb.130.3.503 7542656 PMC2120533

[B43] Febbraio M Silverstein RL CD36: implications in cardiovascular disease Int J Biochem Cell Biol 2007 39 11 2012 2030 10.1016/j.biocel.2007.03.012 17466567 PMC2034445

[B44] Kawaguchi N Sundberg C Kveiborg M Kveiborg M Moghadaszadeh B Asmar M Dietrich N Thodeti CK Nielsen FC Möller P Mercurio AM Albrechtsen R Wewer UM ADAM12 induces actin cytoskeleton and extracellular matrix reorganization during early adipocyte differentiation by regulating beta1 integrin function J Cell Sci 2003 116 19 3893 3904 10.1242/jcs.00699 12915587

[B45] Rodríguez Fernández JL Ben-Ze'ev A Regulation of fibronectin, integrin and cytoskeleton expression in differentiating adipocytes: inhibition by extracellular matrix and polylysine Differentiation 1989 42 2 65 74 10.1111/j.1432-0436.1989.tb00608.x 2633939

[B46] Spiegelman BM Farmer SR Decreases in tubulin and actin gene expression prior to morphological differentiation of 3 T3 adipocytes Cell 1982 29 1 53 60 10.1016/0092-8674(82)90089-7 7105184

[B47] Chiou MJ Wang YD Kuo CM Chen JC Chen JY Functional analysis of mitogen-activated protein kinase-3 (MAPK3) and its regulation of the promoter region in zebrafish DNA Cell Biol 2007 26 11 781 790 10.1089/dna.2007.0613 17999625

[B48] Pagès G Guérin S Grall D Bonino F Smith A Anjuere F Auberger P Pouysségur J Defective thymocyte maturation in p44 MAP kinase (Erk 1) knockout mice Science 1999 286 5443 1374 1377 10.1126/science.286.5443.1374 10558995

[B49] Nutrient Requirements of Poultry NRC 1994 9 Natl. Acad. Press, Washington 4

[B50] Ministry of agriculture of the people's Republic of China Feeding standard of chickens, ICS 65. 020. 30, B 43, NY/T 33–2004 Nutrient Requirements of Yellow-feathered Broiler 2004 China Agriculture Press, Beijing

[B51] Official Methods of Analysis 1990 15th edition. Assoc. Offic. Anal. Chem. Arlington

[B52] Zerehdaran S Vereijken ALJ van Arendonk JAM van der Waaij EH Estimation of genetic parameters for fat deposition and carcass traits in broilers Poult Sci 2004 83 521 525 15109049 10.1093/ps/83.4.521

[B53] Folch J Lees M Sloane Stanley GH: A simple method for the isolation and purification of total lipids from animal tissues J Biol Chem 1957 226 497 509 13428781

[B54] Hatch FT Lees RS Practical methods for plasma lipoprotein analysis Advan Lipid Res 1968 6 1 68 4179999

[B55] Okazaki M Hagiwara N Hara I Hetercrogeneity of human serum high density lipoproteins on high performance liquid chromatography J Biochem 1982 92 2 517 524 7130154 10.1093/oxfordjournals.jbchem.a133959

[B56] Bergmeyer U Bergmeyer J Grassl M Methods of Enzymatic Analysis. Vol. 2. Sample, Regents, Assessment of Results 1974 Academic Press Inc, New York

[B57] Kimura S Iyama S Yamaguchi Y Hayashi S Fushimi R Amino N New enzymatic assay for calcium in serum Clin Che 1996 42 8 1202 1205 8697577

[B58] Kanehisa M Goto S KEGG: kyoto encyclopedia of genes and genomes Nucleic Acids Res 2000 28 1 27 30 10.1093/nar/28.1.27 10592173 PMC102409

[B59] Kanehisa M Goto S Hattori M Aoki-Kinoshita KF Itoh M Kawashima S Katayama T Araki M Hirakawa M From genomics to chemical genomics: new developments in KEGG Nucleic Acids Res 2006 34 D354 D357 10.1093/nar/gkj102 16381885 PMC1347464

[B60] Kanehisa M Araki M Goto S Hattori M Hirakawa M Itoh M Katayama T Kawashima S Okuda S Tokimatsu T Yamanishi Y KEGG for linking genomes to life and the environment Nucleic Acids Res 2008 36 D480 D484 18077471 10.1093/nar/gkm882 PMC2238879

