# Supplementary material for: Identification of differentially expressed genes and pathways for intramuscular fat deposition in pectoralis major tissues of fast-and slow-growing chickens
Source: BMC Genomics. 2012 May 30;13:213. doi: 10.1186/1471-2164-13-213 (PMC3420248; doi:10.1186/1471-2164-13-213)
Supplement: Additional file 7 — Selected qPCR primer sequences and accession numbers. [file 1471-2164-13-213-S7.doc]

| Gene | Sequence | Product size | Accession NO. |
| --- | --- | --- | --- |
| *FABP3* | F:5’-ACGGCCAATTTCGATGAGTACA-3’ R:5’-TCTCTGTGTTCTTGAAGGTGCTAT-3’ | 148bp | AY648562 |
| *RXRA* | F:5’-GCCGCAGTCTAGGAATGATG-3’  R:5’-GGAAGGCAAAGAATGGACACT-3’ | 178bp | NM_002957 |
| *MYH4* | F:5’-AACTTCCAGTCTGCGAGC-3’  R:5’-TCTCCTTTTCTGACTTTCGG-3’ | 144bp | XM_415578 |
| *FGF4* | F:5’-CACAGCGAGAACCGATACAGTC-3’  R:5’-TCCGTTTTTGCTCAGGGC-3’ | 234bp | U14654 |
| *FABP1* | F: 5’-CTCGGGCTTCCTGATGAT-3’  R:5’-AGGTGAGGTCTCCCTTCGT-3’ | 295bp | NM_204192 |
| *PGK1* | F: 5’-CTTAGCAATTCTTGGAGGAG-3’  R:5’-AGTCAACTGGCAAGGTAATC-3’ | 233bp | NM_204985 |
| *PLTP* | F: 5’-ATCAAACCCTCGGGAACCTC-3’  R:5’-TGGAATAGATGCGAAACCGTC-3’ | 184bp | NM_001162406 |
| *RBP7* | F: 5’-TTCCATCCATACCACAAGCACA-3’  R:5’-AGTGAGTCCAGCCCCTGTTCTT-3’ | 179bp | XM_417606 |
| *THRSP* | F:5'-ATCAAGCCCGTGGTGGAGC-3'  R:5'-CTTTGGTGTTTTTGGTGAGGTCG-3' | 184bp | NM_213577 |
| *RBP1* | F: 5’-CGTGGTTGGACTCAGTGGATTG-3’  R:5’-TAGTGCTTGGAGATTTGGTGGC-3’ | 164bp | XM_422635 |
| *ACSF3* | F:5’-CTGCCACTCAGGTCTCATAG-3’  R:5’-TTGTCCCACTCGTGTAGATT-3’ | 121bp | XM_425134 |
| *DCK* | F:5’-AGAGGAGGAAGGAATTGACCTC-3’  R:5’-GTCGAAGACCACATGGCTGT-3’ | 272bp | NM_001079500 |
| *DGAT2* | F: 5’-AATGGGTCCTCACGTTCC-3’  R:5’-TGGTGGTCAGCAGGTTGT-3’ | 237bp | XM_419374 |
| *FABP5* | F: 5’-TACATGAAGGAGCTGGGTG-3’  R:5’-GCTGACAAGGGTCTGAGTTT-3’ | 201bp | NM_001006346 |
| *MYBPC1* | F:5’-ACTATCGCTGTGAGGTTTCC-3’  R:5’-CAAGTTCTCCTGCATCGTCT-3’ | 150bp | BX935207 |
| *GLTPD1* | F:5’-CCACATCCGCAACGTGTAC-3’  R:5’-CCTTCGCTTAGCAAGACCC-3’ | 278bp | XM_417578 |
| *SNX4* | F:5’-TTAGTAATGCTCAAGGGTGCT-3’  R:5’-AAACACCTCAGGAGCTTAAGAC-3’ | 187bp | XM_422107 |
| *FGF7* | F:5’-ATGTGAACTGTTCCAGCCCTG-3’  R:5’-TTGTTTGCTTCTCTCGTCCCT-3’ | 151bp | NM_001012525 |

**Additional file 7.** **Selected qPCR primer sequences and accession numbers**
